# Complete mitochondrial genome of *Hynobius dunni* (Amphibia, Caudata, Hynobiidae) and its phylogenetic position

**DOI:** 10.1080/23802359.2020.1770140

**Published:** 2020-06-01

**Authors:** Takeshi Igawa, Hisanori Okamiya, Hajime Ogino, Masahiro Nagano

**Affiliations:** a Amphibian Research Center, Hiroshima University, Hiroshima, Japan; b Department of Biological Sciences, Tokyo Metropolitan University, Hachioji, Tokyo, Japan; c Faculty of Science and Technology, Oita University, Oita, Oita, Japan

**Keywords:** Mitochondrial genome, amphibian, Caudata, high-throughput sequencing

## Abstract

*Hynobius dunni* is a salamander species of the genus *Hynobius* endemically distributed in eastern Kyushu in southwestern Japan. In this study, we determined the complete mitochondrial genome sequence and clarified the phylogenetic position of this species. The mitochondrial genome was 16,47 bp in length and encoded 13 protein, 2 ribosomal RNA, and 22 transfer RNA genes. Phylogenetic tree based on 13 protein-coding genes revealed that *H. nebulosus* were the most closely related species within the *Hynobius* species. The data identified in this study will be useful for population and conservation genetic studies of *Hynobius* species.

The genus *Hynobius* Tschudi, 1838 is the largest genus in the Asian salamander family Hynobiidae currently including 52 recognized species (Frost 2020). Recent studies led to the discovery of a number of new species within lotic *Hynobius* in Japan, indicating that phylogenetic diversity of these salamanders might still be underestimated (Sugawara et al. 2018; Okamiya et al. 2018; Matsui et al. 2019; Tominaga et al. 2019). The Oita salamander, *Hynobius dunni*, is endemic to eastern Kyushu (Oita, Kumamoto, and Miyazaki Prefectures) in southwestern Japan. This salamander is a lowland lentic breeder and the distribution area is inhabited by human and subject to the effects of anthropogenic activities (Sugawara et al. 2015). Therefore, this species is listed as Endangered (EN) on the IUCN Red List of Threatened Species (International Union for Conservation of Nature 2019).

The phylogenetic studies of this species were previously reported, but were based on partial mt genomes (Sugawara et al. 2015, 2018). Therefore, the complete mt genome of *H. dunni* has not been identified and also, the biological importance of this species has been poorly understood. Here, we sequenced the full mt genome of *H. dunni*, which can help understand its phylogenetic position and evolution of genomes, and provide important information for establishing the conservation strategies.

The *H. dunni* specimen was collected from the Oita University in Oita Prefecture (N33.17°, E131.61°). The voucher specimens (ARCHU-100001) were deposited in the Amphibian Research Center, Hiroshima University. Total genomic DNA was extracted from tail clip of the specimen using DNA suisui-F (Rizo, Tsukuba, Japan) following the manufacturer’s instructions, and the high-throughput DNA sequencing was performed by Bioengineering Lab. Co., Ltd. (Sagamihara, Japan) using DNBSEQ-G400 system (MGI Tech, Shenzhen, China) with a single-end 400 bp sequencing. The obtained raw reads (161,016,329 reads, 64,406,531,600 bp) were trimmed by trimmomatic v0.39 (Bolger et al. 2014) and assembled using MitoZ v2.4a (Meng et al. 2019). Annotation of each gene was manually corrected by comparing with *H. nebulosus* (Zheng et al. 2011).

The complete mitochondrial genome sequence of 16,407 bp was assembled using 33,756 reads of 150,034,667 clean reads with 785 mean coverage depth per nucleotide and deposited in DDBJ (Accession No. LC538211). The resultant genome included 13 protein, 2 ribosomal RNA, and 22 transfer RNA genes. All protein genes started with ATG codon except *COX1* starting with GTG. *COX1*, *COX2*, *ATP8*, *ATP6*, *ND3*, and *ND4L* were terminated by TAA, and *ND1, ND2*, *COX3*, *ND4*, and *Cyt*b were terminated by an incomplete stop codon, T or TA. *ND5* and *ND6* were terminated by TAG and AGA, respectively. The gene arrangement was identical to that observed in other *Hynobius* species (Zheng et al. 2011).

To conduct phylogenetic analysis, the mt genome sequences of 20 salamander species were obtained from NCBI, and *Salamandrella tridactyla* served as outgroup. Phylogenetic position of *H. dunni* in the genus *Hynobius* was revealed by the Bayesian inference tree based on 13 protein genes (Figure 1). Our results show that *H. dunni* was the most closely related to *H. nebulosus*, which supports the previous studies on phylogenetic relationships of *Hynobius* species using partial mt genome sequences (Xia et al. 2012; Sugawara et al. 2018; Okamiya et al. 2018; Tominaga et al. 2019). Our complete mitochondrial genome data of *H. dunni* should be useful for molecular phylogenetic and populational genetic studies on *Hynobius* species, and also contribute to genetic conservation management of *H. dunni* and the other Japanese congenic species.

**Figure 1.**
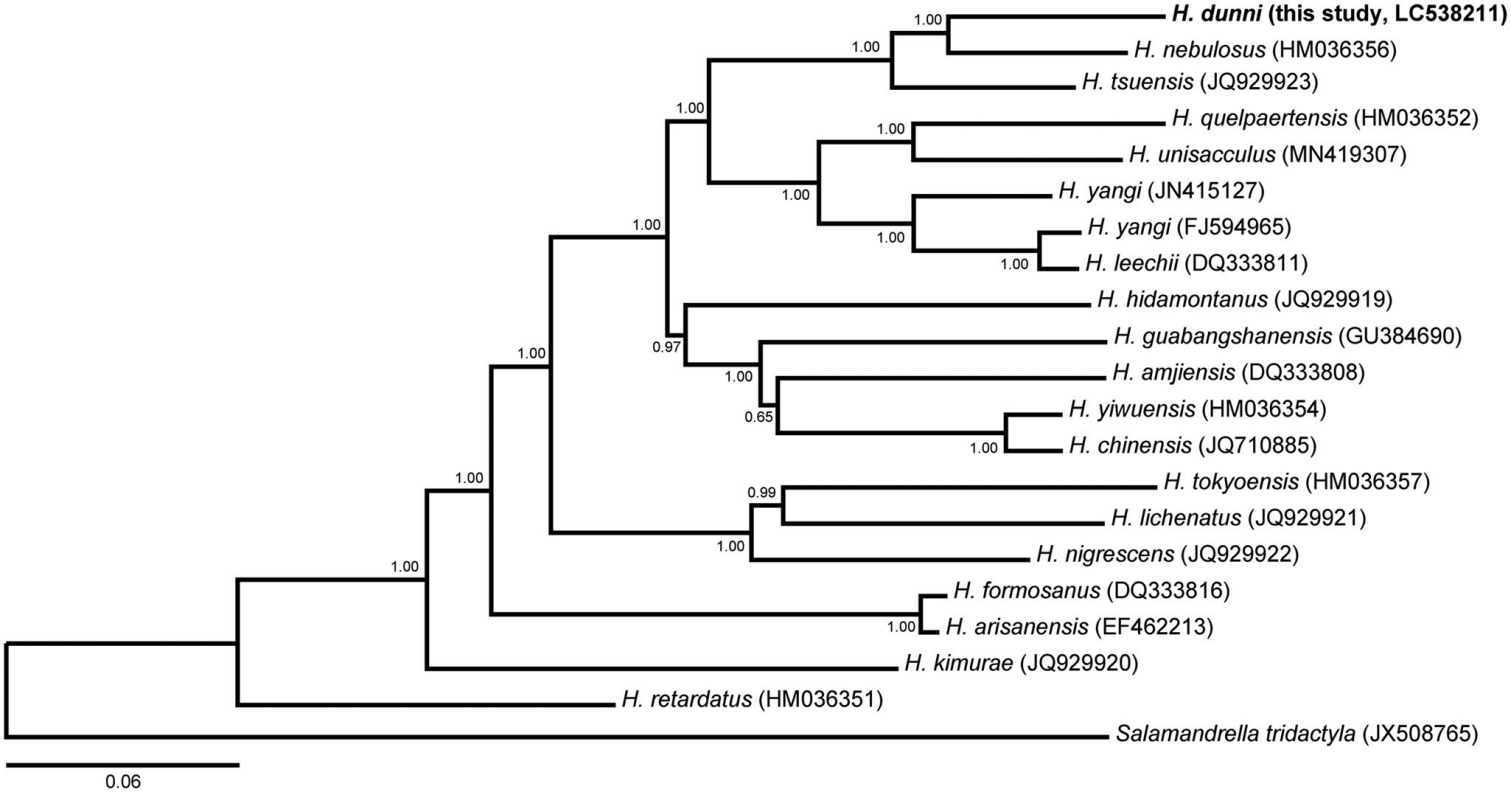
Bayesian inference tree of the genus *Hynobius* based on 13 protein-coding genes of *H. dunni* and the other 18 *Hynobius* species and a *Salamandra tridactyla*. The tree was reconstructed using MrBayes 3.2.7a (Ronquist and Huelsenbeck 2003) with GTR + I + G model selected under Akaike information criterion using Kakusan4 (Tanabe 2007). Analyses were run for three million generations, and trees were sampled every 1000 generations. Convergence among runs was verified by examining the likelihood plots using Tracer 1.7 (Rambaut et al. 2018). The first 25% of trees were discarded as burn-in and the remaining trees were summarized with posterior probabilities at the nodes.

## Data Availability

The data that support the findings of this study are openly available in DDBJ/NCBI/EMBL accession number LC538211 (http://getentry.ddbj.nig.ac.jp/getentry/na/LC538211/).
